# Measurement of Sunlight-Induced Transient Species in Surface Waters

**DOI:** 10.6028/jres.093.047

**Published:** 1988-06-01

**Authors:** Werner R. Haag

**Affiliations:** SRI International, Menlo Park, CA 94025

Sunlight irradiation of natural waters results in absorption of light by dissolved organic and inorganic compounds which then generate a variety of transient species including excited state dissolved organic materials (^3^DOM), singlet oxygen (^1^O_2_), peroxy radicals (ROO·), hydroxyl radicals (HO·), solvated electrons (e^−^_aq_), and superoxide ion (O_2_^−^) [1–12], The transient nature of these species causes both the practical aspects of and philosophy behind their determination to be different from those of conventional, more stable aquatic pollutants. Firstly, transients cannot be concentrated, separated from the water matrix, or the water removed from the light source, and therefore their analysis must be performed by *indirect* kinetic or integrative techniques. Secondly, they do not pose a human health concern because no significant exposure route exists. Ecological effects on lower organisms are possible [13] but none has been documented to date. The primary reason for their interest is that they can affect transformation of natural and manmade compounds. Such transformation can be beneficial, such as in the detoxification of pesticides [141, harmful, such as in the production of toxic peroxidic compounds in the photo-oxidation of crude oils [15], or simply of interest for the understanding of biogeochemical cycles, such as in the cycling of sulfur, nitrogen, and humic materials on geological time scales. Their quantitation allows prediction of environmental fate dynamics, and is of interest in water treatment processes where external sources of transients are added [16,17].

In order to understand how to measure transients, it is necessary to have some understanding of the factors which control their formation and consumption. Figure 1 and table 1 give an overview of some of the main processes involved. The bulk (~99%) of sunlight absorbed by DOM is converted directly to heat. About 1% of the initially formed excited state ^1^DOM undergoes intersystem crossing to the longer-lived ^3^DOM, which transfers the energy to oxygen to form ^1^O_2_, the majority of which, in turn, decays by heating the water. A small fraction of ^3^DOM transfers an electron to oxygen to produce O_2_^−^, which decays by disproportionation and some unknown reactions [12]. A minute fraction of excited state DOM ejects an electron, which is consumed rapidly by dissolved oxygen or possibly by nitrate. The radical cation formed by electron ejection may react with oxygen to form peroxy radicals, or these may be formed by addition of ground state oxygen to excited carbonyls yielding a biradical

$${\;}^{3}R_{2}C=O+O_{2}\to R_{2}C(O\cdot )\text{OO}\to R_{2}C=O+O_{2}+\text{heat}$$

The factors controlling peroxy radical consumption are undefined at present, but may include disproportionation, reaction with DOM, or reversal of O_2_ addition. Hyroxyl radicals are formed mostly by nitrate photolysis and consumed by reaction with DOM in fresh water or bromide ion in seawater.

The data in table 1 include only values measured or estimated thus far and therefore they do not necessarily represent all types of waters. Also, the data are of widely varying accuracy. For example, the formation and loss rates of ^1^O_2_ are accurately known, but the corresponding rates for ROO· and O_2_^−^ are crude estimates. The values for *e*^−^_aq_, HO·, and ^3^DOM appear to be reliable, but there is less data available than for ^1^O_2_ from which to judge accuracy and/or the range of values occurring under a broad variety of conditions. It may be assumed [1], however, that in well-oxygenated waters

$${[}^{3}\text{DOM}]=0.5{[}^{1}O_{2}]$$

and therefore many more [^3^DOM] measurements were inherently estimated from [^1^O_2_] measurements.

Two approaches have been used to measure transient concentrations: derivative and integrative. The first involves following the light-induced rate of loss of a selective trapping agent A, added in low enough concentration that it traps only a small fraction of the transient of interest. This provision is necessary so that A does not perturb the system, i.e., it does not repress the steady state transient concentration, [T]_ss_. Under these conditions

$$\begin{array}{c} -d[A]/dt=k_{r}{\left[T\right]}_{ss}[A]=k_{\exp}[A]\\ {\left[T\right]}_{\text{ss}}=k_{\exp}/k_{r} \end{array}$$

where *k*_r_ is the known second-order rate constant for reaction of A with T, and *k*_exp_ is the experimental, first-order rate constant. Such measurements are simple and give [T]_ss_ directly, but it is crucial to know, at least very crudely, the first-order transient loss rate constant in the absence of A, *k*_d_, in order to calculate the maximum concentration of A which may be used:

$$k_{r}[A]⩽0.1k_{d}$$

An example of the repressive effect of too much trapping agent is shown in figure 2, which shows that ≤ 10 μM benzene should be used when determining [HO·]_ss_ in this particular water sample [8].

The integrative approach involves addition of A in high enough concentration to trap all of the transient as it is formed, and measurement of the formation of a product or loss of a reactant such as oxygen:

$$\begin{array}{c} -d[A]/dt=+d[\text{Product}]/dt={k^{\prime }}_{\exp}\\ {\left[T\right]}_{\text{ss}}={k^{\prime }}_{\exp}/k_{d} \end{array}$$

This method has the drawbacks that it requires precise knowledge of *k*_d_ and that there is always the potential that the high concentrations of A required may affect the lifetime (concentration) of a precursor to the transient of interest. However, the method is more sensitive than the derivative technique and can give quantum yield data which can be used more generally than [T]_ss_ values in making environmental fate predictions. This approach has been used successfully in the determination of [e^−^_aq_]_ss_ [22], and quantum yields of ^1^O_2_ production [3] and total radical production [23].

Clearly important for both methods is that the trapping agent be highly selective for the transient of interest. Testing of selectivity can sometimes be done by quantitation of products, but in some cases products from two transients are so similar that this is not possible. Kinetic tests involve addition of a second solute which modifies the transient lifetime (concentration) in a quantitatively predictable way (table 2). Usually this means adding a competitive quencher or reactant having a known rate constant and seeing if the reduction in rate of loss of the first trapping agent agrees with the predicted factor of *k*_d_/(*k*_d_+*k*_Modifier_[Modifier]). The addition of D_2_O as a diagnostic test for ^1^O_2_ is particularly useful because it selectively causes an increase, rather than decrease, in ^1^O_2_ lifetime and concentrations.

## Figures and Tables

**Figure 1:**
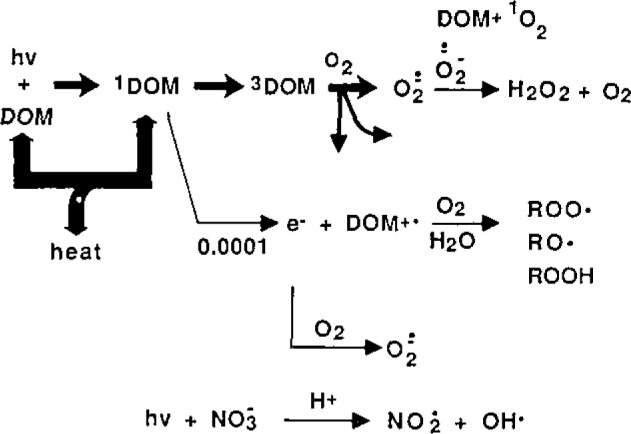
Photochemical pathways for transient formation in surface waters.

**Figure 2:**
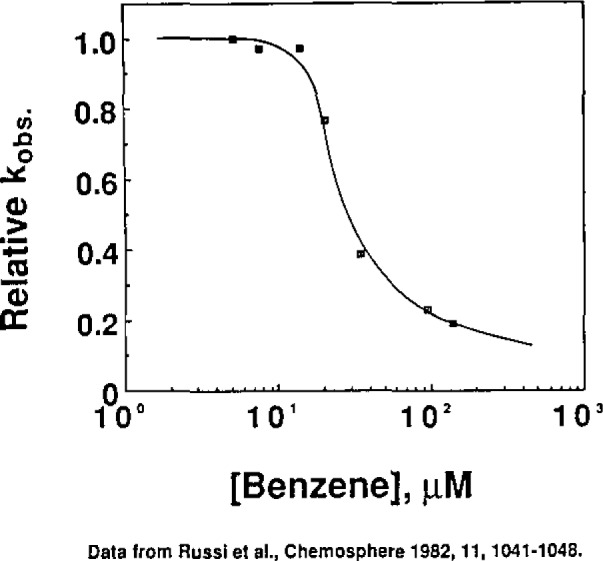
Sensitized benzene hydroxylation in a natural water.

**Table 1:** Kinetic and concentration data for transients in surface water

Transient	Source	Sink^a^	^k^Sink^b^	Formation Rate, M s^−1^	Loss Rate, s^−1^	Surface Concentration, M
^3^DOM	DOM	k_q_(O_2_)	2 × 10^9^ M^−1^s^−1^ [1]	(3–300) × 10^−9^	5 × 10^5^	(1–5) × 10^−13c^
^1^O_2_	DOM	k_q_ (H_2_O)	2.5 × 10^5^ s^−1^ [18]	(3–300) × 10^−9^	2.5 × 10^5^	10^−14^–10^−13d^ 10^−14^–10^−12e^
ROO·	DOM	K_t_[ROO·]^2^ ? k_r_[DOM]	? ?	10^−13^–10^−10^	0.1–1 ?	10^−11^–10^−10^
HO·	NO_3_^−^	k_r_[Br^−^]^d^	1.2 × 10^9^ M^−1^s^−1^ [19]	10^−11^–10^−10^	10^7d^	2 × 10^−19d^
		k_r_[DOM]^e^	2.5 × 10^4^ L mg^−1^s^−1^ [7]		(0.2–2) × 10^5e^	(2–6) × 10^−16e^
e^−^_aq_	DOM	k_r_[O_2_]	2 × 1010 M^−1^s^−1^ [20]	(5–10) × 10^−11^	(0.5–1.5) × 10^7^	(1–2) × 10^−17^
		k_r_[NO_3_^−^]	1 × 10^10^ M^−1^s^−1^ [20]			
O_2_^−^	DOM	k_t_[O_2_^−^]^2^	6 × 10^12^[H^+^] M^−1^s^−1^ [21]	10^−11^−10^−7^ ?	10^−3^−1 ?	10^−9^−10^−8^ ?
		k_r_[DOM]	?			

a The species in brackets or parentheses indicates the interactant and the rate constant subscript indicates the type of interaction: q – energy transfer (quenching), t – termination of two radicals, r – other reactions.

b value of rate constants in previous column

c ≥90 kJ/mol [1]

d Seawater

e Freshwater

**Table 2:** Trapping agents, lifetime modifiers, and typical reactants for transients in surface water

Transient	Trapping Agents	k_r_ M^−1^s^−1^	Lifetime Modifier	k_Modifier_ M^−1^s^−1^	Compounds Affected
^3^DOM	1,3-pentadiene	5 × 10^8^ [1]	O_2_	2 × 10^9^ [1]	nitro-aromatics, dienes
^1^O_2_	furfuryl alcohol dimethylfuran	1.2 × 10^8^ [2]	D_2_^O^	a	furans, imidazoles sulfides, azo-dyes e^−^-rich aromatics
		8.2 × 10^8^ [24]	N_3_^−^	5 × 10^8^ [25]	
ROO·	2,4,6-trimethylphenol	−10^5^ [5]	p-methoxyphenol		phenols, anilines azo-dyes
	p-methoxypheno1	−10^5^		−10^5^	
	p-isopropylphenol	−2 × 10^4^			
HO·	butylchloride	3 × 10^9^ [19]	octanol (organics)	4 × 10^9^ [19]	most organics, nitrite
	benzene	6 × 10^9^ [19]		(1–10) × 10^9^ [19]	
e^−^_aq_	carbon tetrachloride	3 × 10^10^ [20]	O_2_, polyhalo compounds	2 × 10^10^ [20]	polyhalo compounds
	2-chloroethanol	4.1 × 10^3^ [20]		(1–3) × 10^10^ [20]	nitroalkanes ?
O_2_^−^	none identified catechol ? benzidine ?	2.3 × 10^5^ [21] >2.5 × 10^7^ [21]	superoxide dismutase	2 × 10^9^ [21]	none identified benzidines ? catechols ?

a Increases lifetime of ^1^O_2_; k_d_ in H_2_O/D_2_O mixtures is equal to $k_{d}^{H_{2}O}X^{H_{2}O}+k_{d}^{D_{2}O}X^{D_{2}O}$ where X indicates mole fraction and $k_{d}^{H_{2}O}-2.5\times 10^{5}s^{-1}$ and $k_{d}^{D_{2}O}-1.8\times 10^{4}s^{-1}$ [18].
